# Perceptions of transitional care services among patients with percutaneous transhepatic biliary drainage and multicentre health professionals: A qualitative study

**DOI:** 10.1111/hex.13913

**Published:** 2023-11-20

**Authors:** Huan Yu, Xiaomei Wang, Rui Wang, Guoqing Peng, Liyun Gong

**Affiliations:** ^1^ Department of Hepatobiliary Surgery the Second Affiliated Hospital of Chongqing Medical University Chongqing China

**Keywords:** percutaneous transhepatic biliary drainage, qualitative study, transitional care

## Abstract

**Background:**

Patients with percutaneous transhepatic biliary drainage (PTBD) need regular drainage tube care after discharge, and transitional care can help solve this problem. However, few studies have focused on the quality of transitional care, the perceptions of patients with drainage tubes after discharge and those of healthcare professionals.

**Aim:**

This study is aimed at exploring the real experience and perceptions of transitional care services among healthcare professionals and PTBD patients who have been discharged with tubes and at providing references for future transitional care service development.

**Design:**

The study uses a qualitative descriptive design. The reporting method followed Consolidated Criteria for Reporting Qualitative Research guidelines.

**Methods:**

Semistructured interviews were conducted with PTBD patients who had been discharged with tubes and multicentre healthcare professionals using the purpose sampling method. The thematic analysis method was used for analysis.

**Results:**

Thirteen PTBD patients from one hospital and 12 healthcare professionals from three hospitals were interviewed. The analysis of the patient interview data revealed three themes, namely, recognition of the value of transitional care services, patients have some unmet needs and perception of transitional care service pathways. Six subthemes were also identified. The analysis of the interview data of healthcare professionals revealed two themes, namely, harvest and challenges in transitional care services work and expectations for future development of transitional care services. Four subthemes were also identified.

**Conclusions:**

The transitional care of discharged patients with PTBD tubes deserves the attention of clinical workers, and a series of measures should be taken to improve transitional care services.

**Patient/Public Contribution:**

Patients were involved in the formulation of interview questions for this study, and during the interviews, patients presented their suggestions for transitional care services. Healthcare professionals participated in this study as interviewees, and no members of the public were involved in this study.

## 1 INTRODUCTION

Obstructive jaundice is a clinical symptom caused by benign and malignant lesions that occur in or near the biliary tract, such as gallstones, pancreatic head cancer and gallbladder cancer, which prevent bile from flowing into the duodenum and lead to increased levels of bilirubin in the blood. Obstructive jaundice is characterized by abnormal yellow skin, mucosa and sclera, itching and dark urine.^1^, ^2^ In recent years, the incidence of these diseases has been increasing. For example, in 2020 alone, there were nearly 1.51 million new cases of pancreatic, liver and gallbladder cancer worldwide, reflecting an increasing prevalence of obstructive jaundice.^3^ Most patients with malignant obstructive jaundice are in the middle and late stages of the illness when they are diagnosed, making it difficult to carry out surgical treatment, so palliative treatment is the only choice at that time.^4^

Percutaneous transhepatic biliary drainage (PTBD) has been widely used in the clinical treatment of obstructive jaundice due to its advantages of simplistic operation, a high success rate and the rapid relief of symptoms.^5^, ^6^ Patients are generally discharged with tubes after their condition stabilizes to reduce costs and improve hospital efficiency.^7^ Patients with benign obstruction should have their tubes indwelled for at least 12 days.^8^ Patients with malignant obstructive jaundice need to wear the tube until the end of their lives.^4^ Some studies have found that PTBD patients with catheters are prone to complications such as catheter displacement, bleeding and bile leakage after surgery, and the rate of unplanned readmission within 30 days due to drainage tube dysfunction is as high as 63.9%.^9^, ^10^ Therefore, nursing continuity should be strengthened to improve the quality of life of PTBD patients after discharge.

Transitional care has been defined as ‘a set of actions designed to ensure the coordination and continuity of healthcare as patients transfer between different locations or levels of care within the same location’,^11^ including hospital discharge planning, referrals, ongoing follow‐up and the provision of guidance when patients return home or to their community.^12^ Transitional care can be effective in helping patients transition to home or other care settings, and its proper implementation accounts for factors such as the situation of the patient and his or her family, the members of the healthcare team and the relevant environmental and social conditions.^13^ Understanding the patient experience and actual needs is the basis of transitional care and the premise of implementing patient‐centred care.^14^ Understanding the views of healthcare providers can help further the implementation of transitional care. However, the majority of the current studies on transitional care for PTBD patients are intervention studies exploring the effects of different transitional care modalities. A very small number of studies have focused on the quality of transitional care services for PTBD patients and explored the experiences and perceptions of PTBD patients and healthcare professionals regarding the current transitional care services through a qualitative study.^15^ Therefore, by interviewing both discharged PTBD patients and healthcare professionals, this study aimed to explore their perceptions regarding the current transitional care services to provide references for establishing better transitional care for PTBD patients to improve their quality of life.

## 2 METHODS

### 2.1 Design

A descriptive qualitative research design is adopted for this study.^16^ This methodology is appropriate for identifying and understanding a phenomenon, a process or the perspectives of participants. Moreover, this study used the Consolidated Criteria for Reporting Qualitative Research^17^ (COREQ) as a guide in the reporting phase to explicitly and comprehensively report the study results (see Appendix S1).

### 2.2 Selection of participants

From January to March 2023, a purposive sampling method, which includes criterion sampling and maximum variation sampling, was used in this study to select participants from among PTBD patients and healthcare professionals in the Department of Hepatobiliary Surgery of a 3500‐bed university teaching hospital in Chongqing, China. In addition, to provide a more thorough grasp of the opinions of healthcare professionals, members of the nursing staff from other hospitals in Chongqing were also enlisted as participants in this study, which was conducted from a multicentre perspective.

The inclusion criteria for patients with PTBD tubes were as follows: (1) patients were ≥18 years; (2) patients spent 1 month or longer with a PTBD tube and had received transitional care services before returning to the hospital for the subsequent phase of therapy or drain care; (3) patients exhibited sufficient cognitive and communication ability to provide informed consent and participate in the interviews. The exclusion criteria consisted of the presence of severe disease or complications in patients.

The inclusion criteria for healthcare professionals were as follows: (1) doctors and nurses had to have been engaged in medical or nursing work with a specialization in hepatobiliary surgery in tertiary hospitals for 5 years or more; (2) these medical professionals must have experience conducting transitional care services for PTBD patients, specific elements of transitional care services including establishing a transitional care team (doctors, nurses, etc.), collaborating with team; assessing patients and families' situation to create plans for transitional care; patients and families health education at the time of discharge (including how to take care of drains and skin, how to take medication, when to schedule follow‐up appointments, etc.); maintaining relationships with patients, following up with regular phone calls after the patient is discharged from the hospital; and using the internet and other resources to answer questions and offer guidance to patients, promoting care continuity; (3) healthcare professionals must have a bachelor's degree or above. Exclusion criteria consisted of medical personnel who were currently undergoing training.

The sample size calculation followed the principle of data saturation, which means that recruitment ceased when no new information was obtained through interviewing additional participants.^18^

### 2.3 Recruitment procedure

The study team travelled to the ward and explained the purpose of the interview to those healthcare professionals who met the inclusion criteria in a face‐to‐face manner, obtained their informed consent and then scheduled the interviews. In addition, the study team introduced the inclusion and exclusion criteria for patients to these healthcare professionals, who then referred patients who might meet the criteria to the study team. After eligibility screening by the study team, the purpose and content of the study were explained to patients who met the criteria in a face‐to‐face manner, and after obtaining their informed consent, the interviews were scheduled.

### 2.4 Data collection

Face‐to‐face interviews were conducted in an independent and quiet office, and only the interviewer, recorder and interviewee were permitted to be present in the room. The interviews were conducted by H.Y., a nursing graduate student who carefully studied the pertinent methods of qualitative interview and repeatedly practised and confirmed her mastery of the skills needed to conduct qualitative interviews under the guidance of a nursing professor (XM.W.). After obtaining the participant's consent and having the informed consent form signed, the researcher initially documented the participant's basic information, such as age and time with tube/work. Then, formally begin the interview in accordance with the interview outline.

The interview outline was initially developed based on the relevant literature. Then, based on the expert's research orientation, qualifications and willingness to provide guidance, a clinical nursing expert with a senior title who specializes in surgical nursing and has some experience in qualitative studies was consulted for her opinions regarding further revisions (such as whether the interview questions were reasonable, whether they could accurately reflect the purpose of the study, and whether they should be presented in a different order). After the preinterviews with two participants, the outlines were amended to create formal outlines (see Appendix S2).

The entire interview process was audio recorded using a mobile phone. At the same time, important information, such as the participants' expression and tone were recorded in writing by GQ.P. Most importantly, during the interview, participants were not interrupted at will, the interviews remained quiet and listened actively, the order of the interview questions was adjusted according to the participant's answers and the details were pursued in a timely manner when necessary, with any unclear or mentioned crucial points being confirmed through the use of repetition, follow‐up questions or counterquestions.

### 2.5 Data analysis

Within 24 h after the interview, the researcher converted the recording into text, and other members of the research group checked it and returned it to the participants for reconfirmation. Two research members (H.Y. and LY.G) used Nvivo 11.0 software and thematic analysis method for analysis^19^: (1) familiarizing with data: all interview data were read in detail, noting down initial ideas; (2) generating initial codes: extract statements related to transitional care experiences and perceptions, encode significant statements and recurring ideas; (3) searching for themes: collating codes into potential themes and gathering all data relevant to each potential theme; (4) reviewing themes: checking the themes in relation to the coded extracts and the entire data set; (5) defining and naming themes: ongoing analysis to refine the specifics of each theme, generating clear definitions and names for them; (6) producing the report: select vivid and compelling excerpts, connect the analysis to research questions and literature, and produce scholarly reports of the analysis.

### 2.6 Ethical considerations

This study was reviewed and approved by the Ethics Committee of the hospital (ethical review no. 154, 2022). All participants participated voluntarily and signed informed consent. During the interview, participants could terminate the interview at any time.

### 2.7 Reflexivity and rigour

The study team members were all female, and the team included one professor, one master of nursing and three nursing graduate students who had taken a course in qualitative research. The researchers were students and not involved in the clinical work of the ward, so no established prior relationships existed between the interviewer and the interviewees to ensure the quality of the interviews.

The study team took the following actions to guarantee the validity of the findings. During the data collection phase, interviews were conducted on the basis of a carefully revised semistructured interview outline, and the entire interview process was recorded. During the data analysis stage, the transcribed text was returned to the participants for confirmation, and two researchers immersed themselves in the data for an extended period and worked on coding and analysing the information. In addition, the researchers maintained a reflective and engaged in team discussions to avoid preunderstanding influencing the study.

## 3 RESULTS

### 3.1 Characteristics of participants

Finally, 13 PTBD patients from one hospital and 12 healthcare professionals from three hospitals were interviewed, and none of the participants dropped out of the interview. The duration of each interview is 10–50 min. The average age of the patient was (49.77 ± 12.51) years old, the average duration of catheterization was (7.73 ± 12.63) months, the average age of the healthcare staff was (37.25 ± 5.26) years and the average years of working in the specialty were (11.65 ± 5.46) years. Details are shown in Tables 1 and 2.

**Table 1:** General data of PTBD patients (*N* = 13).

Number	Gender	Age	Educational background	Drainage tube time	Care mode of PTBD drainage tube
P1	Male	33	Junior middle school	3 months	Home care
P2	Male	47	Primary school	1 month	Hospital care
P3	Female	67	Primary school	10 months	Home care
P4	Female	49	Junior middle school	4 years (lifetime tube)	Home care + community care
P5	Male	48	Junior middle school	4 months	Home care
P6	Male	63	Junior middle school	8 months	Home care
P7	Male	40	Junior middle school	1 month	Home care
P8	Male	59	Primary school	1 year	Home care
P9	Female	59	Senior middle school	1 month	Home care
P10	Female	22	Senior middle school	6 months	Home care + community care
P11	Male	58	Primary school	2 months	Home care
P12	Female	53	Junior middle school	3 months	Home care
P13	Male	49	Junior middle school	2 months	Home care

Abbreviation: PTBD, percutaneous transhepatic biliary drainage.

**Table 2:** General data of healthcare staff (*N* = 12).

Number	Gender	Age	Educational background	Professional title	Position	Years of specialist work
N1	Female	39	Undergraduate	Supervisor nurse	Specialist nurse	21
N2	Female	31	Undergraduate	Nurse Practitioner	General clinical nurse	9
N3	Female	47	Undergraduate	Supervisor nurse	Head nurse	20
N4	Male	36	Doctor of Medicine	Deputy chief physician	Clinician	7
N5	Female	35	Undergraduate	Supervisor nurse	Specialist nurse	13
N6	Female	35	Undergraduate	Supervisor nurse	General clinical nurse	5
N7	Female	42	Undergraduate	Nurse in charge	Deputy head nurse	18
N8	Female	45	Undergraduate	Deputy chief nurse	Deputy head nurse	13
N9	Female	32	Undergraduate	Supervisor nurse	Teaching team leader	10
N10	Female	32	Undergraduate	Supervisor nurse	Quality management team leader	11
N11	Male	34	Doctor of Medicine	Attending physician	Clinician	5
N12	Male	39	Doctor of Medicine	Deputy chief physician	Clinician	9

### 3.2 Themes from patient interviews

Table 3 shows the themes and codes derived from the patient interviews. Please see Appendix S3 for a supplementary table with all the quotes.

**Table 3:** Results of patient interview analysis.

Themes	Subthemes	Codes
Recognition of the value of transitional care services	Appreciate the positive attitudes of healthcare professionals	Patience and carefulness
		Timely responses
	Benefit from transitional care services	Feeling positive about regular follow‐ups
		Solving the problems related to self‐care
Patients have some unmet needs	Potential psychological needs	Excessive worry about drain prolapse and infection
		Self‐image disorders affecting social interaction
		Irritation with the inconvenience of living with a tube
	Knowledge needs for disease care	Management of drainage tube emergencies
		Personalized lifestyle guidance (diet, exercise)
Perception of transitional care service pathways	Preferred transitional care model for the hospital‐to‐home transition	Savings in time and money
		Availability issues with drainage tube care services
	Preferred network information guidance and telephone follow‐up	Convenient and swift
		Network guidance visibility

#### 3.2.1 Theme 1: Recognition of the value of transitional care services

Interviews revealed that most patients were satisfied with the transitional care services they received and that the attitude and effectiveness of the services were crucial factors.

##### Appreciate the positive attitudes of healthcare professionals

Attitude is one of the key factors influencing patient satisfaction with nursing care. The harmonious relationship between nurses and patients is conducive to the practice of nursing. Most patients reported being deeply impressed by the professional attitude of the doctors and nurses, the healthcare staff responded quickly to their questions, and this positive attitude made them feel supported. ‘You always have a smile, and you are very patient in helping me to change the medicine and care, I am very satisfied with your work’ (P5).

##### Benefit from transitional care services

Previously, patients often had problems with drain care after being discharged, and the transitional care service enabled patients to acquire relevant knowledge under the guidance of healthcare professionals, facilitating the implementation of self‐care after discharge, effectively providing physiological benefits to patients and promoting patient acceptance of the service. Moreover, the pattern of regular follow‐up visits makes the patients feel psychologically satisfied. ‘Every time I was discharged from the hospital, you would call to determine how I was doing. With your care and help, I can take better care of myself now!’ (P8).

#### 3.2.2 Theme 2: Patients have some unmet needs

This study found that patients need in‐depth help from healthcare providers in psychological and disease care knowledge.

##### Potential psychological needs

Most patients reported that carrying a drain relieved the symptoms of their disease, but it also interfered with their daily life, changed some of their habits and made them less comfortable and irritable. Some patients also developed anxiety and even experienced negative emotions, such as excessive worry about problems with their drain, a lack of self‐confidence and fear of socializing. The heavy psychological burden experienced by patients reflects their underlying psychological needs and requires attention from healthcare professionals. ‘Sometimes it is quite annoying to have a drain, I cannot shower comfortably, I have to sleep more carefully, and I go out less often’ (P13).

##### Knowledge needs for disease care

Patients are generally discharged from the hospital with instructions on drain care and medication provided by healthcare professionals. However, some patients mentioned that the discharge instructions were somewhat broad and that they would like more personalized life guidance (diet, exercise). Other patients reported feeling as if they understood what the nurses were telling them when they were discharged from the hospital but were not quite sure what to do after they were discharged. In addition, some patients realized that they still had some nursing knowledge needs when they encountered a nursing problem after discharge, such as the management of drain emergencies. These reports indirectly reflect that discharge guidance is not sufficiently comprehensive and specific and that attention should be given to facilitating patients' knowledge acquisition. ‘One time my drain fell out, but the doctor probably did not think about that and did not tell me, so when the tube fell out, I put it in myself and it hurt my stomach’ (P1).

#### 3.2.3 Theme 3: Perception of transitional care service pathways

Patients who had experienced transitional care services exhibited some preference for the way in which transitional care services were delivered.

##### Preferred transitional care model for the hospital‐to‐home transition

In this study, it was found that most of the patients preferred receiving home care for their drains after discharge due to their financial situation. Service accessibility is also a factor considered by patients. Some community hospitals have not yet launched drain care services, and patients living in those communities must travel to the outpatient clinics of large hospitals to receive drain care, which is both time‐consuming and energy‐consuming. ‘I prefer that doctors and nurses teach me how to care for the pipes, because my home is far away from the town, and it costs some money every time’ (P5).

##### Preferred network information guidance and telephone follow‐up

The network information technology guidance method with microblogging, public numbers and other carriers is convenient and conveys visual, rich and vivid content, and the ease with which this content can be understood facilitates the majority of patients accepting follow‐up care. In most cases, healthcare professionals follow more than one form of practice and need to account for patients who do not have access to the internet, so telephone follow‐ups are also acceptable to patients. ‘You can use the internet, we can learn on it, it is all very convenient now, otherwise we will forget after you have talked about it’ (P9).

### 3.3 Themes of healthcare professionals interviews

Table 4 shows the themes and codes for healthcare professionals. Please see Appendix S3 for a supplementary table with all the quotes.

**Table 4:** Results of healthcare professionals interviews analysis.

Themes	Subthemes	Codes
Harvest and challenges in transitional care services work	Bringing a positive emotional experience	Appreciation and trust from the patients
		Improvements in patient drain care outcomes
		The improvement of one's professional ability
	Existing difficulties and challenges	Insufficient human resources for healthcare professionals
		Process and education guidance need to be optimized
		Lack of support from a well‐developed information system
Expectations for future development of transitional care services	Hospital and departmental level	Human resource allocation and training
		Support from facilities and related norms
	Social level	Anticipation of community participation

#### 3.3.1 Theme 1: Harvest and challenges in transitional care services work

Healthcare professionals described their views about transitional care from a wider perspective.

##### Bringing a positive emotional experience

Participants reported that they gained a sense of achievement from building harmonious relationships with their patients, implementing transitional care services for their patients, and improving drain care outcomes. ‘I have taken on the role of missionary and mentor in my work, and I feel that I have realized the value of my work through my own efforts’ (N3).

Trust and gratitude from patients also provide them with positive motivation. In addition, healthcare professionals believe that the process of serving patients is also a process of self‐learning, through which they gain experience and improve themselves. ‘A patient wrote me a thanks letter before. In fact, these are what I should do, but her sincerity touched me. I will always remember this trust and strength’ (N4).

##### Existing difficulties and challenges

Numerous participants mentioned the issue of human resources, and one healthcare worker reported having to manage multiple patients and limited time and energy, which to some extent led to a shorter time needed for the implementation of transitional care. ‘Human resources are a big problem, there is a lot of work every day, and sometimes I feel as if it is almost enough to do a good job in the basic operations for patients. Transitional care needs to spend more time and energy to educate and contact patients’ (N9).

In addition, although a system for transitional care management is now in place and transitional care is implemented through WeChat, participants said that they found that, in practice, the transitional care process and didactic guidance needed to be further harmonized and further deepened to avoid compromising patients' grasp of knowledge. ‘Sometimes the patient will ask us what they should do to change the drainage tube themselves, and we will guide them. However, we do not have uniform guidelines, and if you do not do a good job, you might get in trouble’ (N2).

Currently, nursing continuity relies on the support of an information platform. The existing WeChat platform can be used to realize basic functions such as video guidance and communication, but doctors and nurses have expressed the need to develop a more complete and feature‐rich information platform for the support of transitional care, and this is a challenge. ‘It is good to have a WeChat platform, so that patients can ask questions directly, and we can push nursing knowledge, but if I want to do more, such as monitoring the patient's postdischarge diversion, the WeChat group chat approach does not work’ (N12).

#### 3.3.2 Theme 2: Expectations for future development of transitional care services

Participants expressed their expectations for future developments, as they all wanted to make transitional care better.

##### Hospital and departmental level

Participants would like to see more invested in human resources, which requires decision‐making on the part of managers to allocate and manage human resources more appropriately. Some participants suggested that every charge nurse should be involved in transitional care. In addition, systems and norms should be improved, and team members should master the knowledge and collaborate with each other to better provide transitional care for patients. ‘We must review the literature, develop scientific guidelines, improve follow‐up systems and processes, and pass on the right knowledge to patients and families’ (N5).

As the demand for healthcare services is increasing, participants said that they would like to have a strong information platform to ensure the continuity and privacy of services to achieve information sharing, which is difficult to achieve through individual efforts alone. ‘I hope that we can introduce a smarter platform to help us build personal health records for discharged patients and implement personalized management’ (N10).

##### Social level

At present, it is mainly the nursing staff in hospitals who are responsible for implementing transitional care, and the out‐of‐hospital support is relatively weak. The participants expressed a desire for more social‐level support in the future through community involvement, for example. It is very meaningful. ‘I think it is important to work with the community because the general trend is that more and more patients are being discharged with tubes. If the community develops, then the resources of the large hospital can be properly allocated, and patients can save time and energy’ (N2).

Participants mentioned that if an effective hospital–community interface is to be realized, it is important to account for the reality of distance, how to effectively communicate and collaborate, and training and quality monitoring of the community staff. ‘The problem we are facing is now that we have some medical joint units, the patients are relatively scattered, and many units have not established contact with them’ (N4).

## 4 DISCUSSION

In this study, both patients and healthcare professionals reported having had positive experiences with transitional care. We can find one reason for this is the positive interaction between patients and healthcare professionals, who mutually support each other. The other is the reality that transitional care genuinely has helped the patient, which is the result that healthcare professionals want to achieve. What has been done well should be continued, and improvements are made in response to the identified problems. Negative psychological emotions, such as worry, irritability and the fear of socializing, are profound experiences of PTBD patients following their discharge from the hospital, and these experiences can seriously affect their quality of life. It is suggested that healthcare professionals should pay more attention to the psychological state of PTBD patients, which is consistent with the findings of Mo Wei's study.^20^ For patients with benign obstructive jaundice, PTBD can facilitate the rapid relief of jaundice and infection symptoms and provide a method for follow‐up treatment.^21^ The relatively short time these patients had spent on the tube may have contributed to their positive attitude. However, for patients with malignant obstructive jaundice, PTBD is a palliative treatment that can only relieve obstructive jaundice but has no effect on tumours invading bile ducts and thus cannot completely cure the disease.^22^ Patients need to endure the pain of the disease itself and the discomfort caused by the drainage tube. In this study, it was found that although patients with long‐term tubes have gradually become accustomed to living their lives with tubes, they also have a need for psychological care. Carrying the drainage tube for a long time causes serious interference in daily life and work, resulting in psychological disorders.^23^ The negative emotion of some patients is related to a lack of correct cognition regarding the pipeline, which may lead to changes in medical compliance behaviour and consequently affect treatment outcomes.^24^ Therefore, it is recommended that the transitional care team should strengthen the health education and humanistic care of patients, change patients' incorrect cognition, and give patients confidence and support to alleviate their adverse psychological emotions.

PTBD can successfully enable biliary decompression, but because it is an invasive intervention with potential complications, blockage, dislocation and cholangitis are often encountered in long‐term catheter therapy.^25^ Standardized PTBD drainage tube care helps to reduce complications, ensure the effectiveness of interventional surgery and improve patients' quality of life.^21^ Rattanakanlaya found that the quality of discharge guidance for PTBD patients was positively correlated with their discharge readiness, and a high degree of discharge readiness indicates that patients are ready for home care, have a better grasp of the knowledge of disease pipeline nursing and have a higher compliance and self‐care ability, which could effectively improve their quality of life outside the hospital.^15^ Therefore, the transitional care team should pay attention to the quality of discharge guidance and ensure that it is provided according to the needs of patients so that patients and their families can master the nursing knowledge of drainage tubes during hospitalization and reduce the pressure of transitional care after discharge.^26^ However, the patient's age, education level, learning and acceptance ability, health literacy, mental state and physical condition can all affect discharge guidance.^27^ The feedback method is recommended for health education.^28^ Daoqiong Huang et al.^29^ have shown that this form of information feedback helps patients master self‐care skills for PTBD drainage tubes. At the same time, the discharge guidance provided by healthcare professionals should be combined with the specific situation of patients, targeted guidance for patients and repeated self‐care knowledge.

Many participants in this study mentioned the problem of insufficient human resources, and reasonable human resource allocation is an important guarantee for the development of medical and healthcare activities. Nurses are now an important part of the successful implementation of transitional care.^30^ According to the National Nursing Development Plan (2021–2025) issued by the National Health Commission of China,^31^ by the end of 2020, there were more than 4.7 million registered nurses in China, which represents an increase of 45% over those in 2015. The number of registered nurses per 1000 population reached 3.34. However, compared with the economic and social development and the growing health needs of the people, the number of nurses in China is relatively insufficient and the distribution is still uneven. Nurses' choice of occupation is affected by salary and welfare, working hours, mode, intensity and environment. In addition to the national government strengthening the creation of nurse talent teams and the provision of welfare protection from macro policies, medical institutions should also implement reasonable human resource allocation strategies.^32^

It is critical that the nurses who provide care to patients have the best medical knowledge,^21^ because their nursing behaviour and quality can affect patient outcomes. A national survey in the Netherlands found that nurses working in hospital gastrointestinal surgery had a low overall knowledge of PTBD placement procedures and catheter care, with half of the nurses reporting that their existing knowledge was insufficient.^33^ In China, a survey on PTBD drainage tube care for patients with malignant biliary obstruction in 130 hospitals showed that there were obvious deficiencies in drainage tube care, such as the fixing of biliary drainage tubes, the nursing of puncture points and the connection of drainage bags.^34^ Notably, in 2022, Chinese scholars released the expert consensus on the drainage nursing of PTBD.^35^ It is pointed out that the health education of PTBD patients should include the necessity of retaining the PTBD drainage tube, proper fixation and effective drainage, the observation and recording of drainage fluid, clamp tube nursing, skin care, diet and nutrition, activity guidance and requests for follow‐up visits. Nursing managers should pay attention to the frontier progress of the discipline, apply the best evidence to the clinic and formulate optimal scientific education guidance. At the same time, the nursing staff should improve the standardized training of drainage tube nursing so that they can better educate patients and their families and ensure that drainage tubes are maintained properly following patient discharge. The state also encourages institutions to actively provide transitional care, continuously improve the quality of care and strive to build a system of nursing norms and technical standards from an evidence‐based perspective that accounts for clinical needs to promote the development of high‐quality nursing.^31^ In this study, healthcare professionals mentioned that patient satisfaction and drainage tube nursing outcomes could be used to evaluate transitional care quality. These two indices fall under the result quality, and it is necessary to establish a scientific and feasible structure‐process‐result quality evaluation index system to standardize nurses' behaviour and improve the quality of transitional care for PTBD patients.^36^

At present, there are few studies on hospital‐community communication for PTBD patients. Although healthcare professionals expressed a desire for community involvement, they believe that it is meaningful to cooperate with the community to carry out transitional care, it is difficult to establish the hospital–community‐family ternary linkage form due to the lack of an information platform between the hospital and the community. This difficulty affects the seamless connection between treatment and nursing inside and outside the hospital and the maximum utilization of medical resources.^12^ Effective online communication with patients through mobile platforms can improve patients' quality of life and is unconstrained by time and space.^37^ Therefore, it is necessary for managers to strengthen the construction of information systems and develop richer functions to provide support for the construction of three‐way linkage transitional care model.

## 5 STRENGTHS AND LIMITATIONS

In the study, interviews were conducted with patients with benign and malignant obstructive jaundice with PTBD tube, doctors, clinical nurses and care managers using maximum variation sampling. This helped us to comprehensively and thoroughly explore the relevant information from multiple perspectives. Moreover, we used several measures to ensure the rigour of the study, such as a carefully revised semistructured interview outline, detailed records of sample selection and the data collection and analysis process.

However, there are some methodological limitations to our study as well. First, due to limited resources available to the study team, the participants were all from Chongqing, China. Thus, they could not represent the status quo of transitional care for PTBD patients in hospitals across the country. The different economic levels and medical environments restrict the generalizability of the results, so future studies should conduct research using a more diverse sample. Second, some interview questions were related to participants' previous transitional care experiences, for which they needed to recall past situations. Therefore, potential bias may have been introduced due to the unreliability of memory. In addition, although we returned the transcribed data to the participants for face‐to‐face confirmation, the data analysis results were not discussed with the participants because of limited resources and limited availability of the participants' time and energy. This lack could limit the transferability of the results.

## 6 CONCLUSION

Both PTBD patients and healthcare professionals had positive experiences with transitional care. However, the transitional care needs of PTBD patients were not fully met, and transitional care management needs to be improved. First, we should strengthen the construction of transitional care team and allocate more human resources reasonably. Second, we should improve the relevant facility system and establish a standardized process. In the process of implementation, we should pay attention to the quality of discharge guidance and the psychological feelings of patients to improve the quality of life of patients following discharge.

## 7 RELEVANCE TO CLINICAL PRACTICE

This study analysed views of PTBD patients and healthcare professionals regarding the transitional care services, and proposed corresponding countermeasures, which possess a certain guiding significance for clinical practice, such as strengthening team collaboration and extension care facility process construction, while considering the needs and feedback of patients to better implement the transitional care services for PTBD patients in clinical practice.

## AUTHOR CONTRIBUTIONS

Huan Yu was involved in interviewing the participants, data analysis and writing the initial draft. Xiaomei Wang and Rui Wang were involved in study design and surveillance, and made the interview guide. Guoqing Peng was responsible for taking notes during the interview and sorting out data. Liyun Gong was involved in text transcription and analysis. All authors read and approved the final manuscript.

## CONFLICT OF INTEREST STATEMENT

The authors declare no conflict of interest.

## ETHICS STATEMENT

This study was reviewed and approved by the Ethics Committee of the hospital. All patients participated voluntarily and signed informed consent.

## Supporting information

Supporting information.

Supporting information.

Supporting information.

Supporting information.

Supporting information.

Supporting information.

## Data Availability

The full interview data are not publicly available due to privacy or ethical restrictions. The data that support the findings of this study are available from the corresponding author upon reasonable request.

## References

[1] Asare O , Osei F , Appau AY , et al. Aetiology of obstructive jaundice in Ghana: a retrospective analysis in a tertiary hospital. J West Afr Coll Surg. 2020;10(3):36‐39. 10.4103/jwas.jwas_46_21 PMC9202603 35720953

[2] Soares PFC , Gestic MA , Utrini MP , Callejas‐Neto F , Chaim EA , Cazzo E . Epidemiological profile, referral routes and diagnostic accuracy of cases of acute cholangitis among individuals with obstructive jaundice admitted to a tertiary‐level university hospital: a cross‐sectional study. Sao Paulo Med J. 2019;137(6):491‐497. 10.1590/1516-3180.2019.0109170919 32159634 PMC9754274

[3] Sung H , Ferlay J , Siegel RL , et al. Global Cancer Statistics 2020: GLOBOCAN estimates of incidence and mortality worldwide for 36 cancers in 185 countries. CA Cancer J Clin. 2021;71(3):209‐249. 10.3322/caac.21660 33538338

[4] Zerem E , Imširović B , Kunosić S , Zerem D , Zerem O . Percutaneous biliary drainage for obstructive jaundice in patients with inoperable, malignant biliary obstruction. Clin Exp Hepatol. 2022;8(1):70‐77. 10.5114/ceh.2022.114190 35415254 PMC8984794

[5] Chen GF , Yu WD , Wang JR , Qi FZ , Qiu YD . The methods of preoperative biliary drainage for resectable hilar cholangiocarcinoma patients: a protocol for systematic review and meta analysis. Medicine. 2020;99(21):e20237. 10.1097/MD.0000000000020237 32481299 PMC7249990

[6] Nikolic I , Radic J , Petres A , et al. The clinical benefit of percutaneous transhepatic biliary drainage for malignant biliary tract obstruction. Cancers. 2022;14(19):4673. 10.3390/cancers14194673 36230596 PMC9563508

[7] Kim MU , Lee Y , Lee JH , et al. Predictive factors affecting percutaneous drainage duration in the percutaneous treatment of common bile duct stones. PLoS One. 2021;16(3):e0248003. 10.1371/journal.pone.0248003 33651811 PMC7924786

[8] Sochnieva AL . Optimum duration of percutaneous transhepatic cholangiodrainage in common bile duct diseases complicated by obstructive jaundice. Wiad Lek. 2020;73(9 cz. 2):1915‐1925. 33148834

[9] Sarwar A , Hostage Jr. CA , Weinstein JL , et al. Causes and rates of 30‐day readmissions after percutaneous transhepatic biliary drainage procedure. Radiology. 2019;290(3):722‐729. 10.1148/radiol.2018180279 30599096

[10] Subramani VN , Avudaiappan M , Yadav TD , et al. Outcome following percutaneous transhepatic biliary drainage (PTBD) in carcinoma gallbladder: a prospective observational study. J Gastrointest Cancer. 2022;53(3):543‐548. 10.1007/s12029-021-00655-5 34173180

[11] Coleman EA , Boult C . Improving the quality of transitional care for persons with complex care needs: position statement of the American Geriatrics Society Health Care Systems Committee. J Am Geriatr Soc. 2003;51(4):556‐557. 10.1046/j.1532-5415.2003.51186.x 12657079

[12] Gao Y , Tang X , Wen Y , Qian D , Pan X , Zhang L . Effects of the hospital‐community‐family ternary linkage continuous nursing model on compliance, cognitive function, resilience, and quality of life for children with epilepsy: a retrospective study. Transl Pediatr. 2022;11(2):239‐248. 10.21037/tp-22-21 35282024 PMC8905107

[13] Shahsavari H , Zarei M , Aliheydari Mamaghani J . Transitional care: concept analysis using Rodgers' evolutionary approach. Int J Nurs Stud. 2019;99:103387. 10.1016/j.ijnurstu.2019.103387 31442782

[14] Raj M , Platt JE , Anthony D , Fitzgerald JT , Lee SYD . What does “Patient‐Centered” mean? Qualitative perspectives from older adults and family caregivers. Gerontol Geriatr Med. 2021;7:233372142110176. 10.1177/23337214211017608 PMC8145610 34104684

[15] Rattanakanlaya K , Vuttanon N , Noppakun L , Sangwattanarat W , Boonyu N , Iamruksa S . Readiness for hospital discharge post‐initial invasive percutaneous transhepatic biliary drainage: a mixed‐methods study. Heliyon. 2023;9(5):e15341. 10.1016/j.heliyon.2023.e15341 37144202 PMC10151257

[16] Bradshaw C , Atkinson S , Doody O . Employing a qualitative description approach in health care research. Glob Qual Nurs Res. 2017;4:233339361774228. 10.1177/2333393617742282 PMC5703087 29204457

[17] Tong A , Sainsbury P , Craig J . Consolidated criteria for reporting qualitative research (COREQ): a 32‐item checklist for interviews and focus groups. Int J Qual Health Care. 2007;19(6):349‐357. 10.1093/intqhc/mzm042 17872937

[18] Hennink MM , Kaiser BN , Marconi VC . Code saturation versus meaning saturation: how many interviews are enough? Qual Health Res. 2017;27(4):591‐608. 10.1177/1049732316665344 27670770 PMC9359070

[19] Saunders CH , Sierpe A , Von Plessen C , et al. Practical thematic analysis: a guide for multidisciplinary health services research teams engaging in qualitative analysis. BMJ. 2023;381:e074256. 10.1136/bmj-2022-074256 37290778

[20] Wei M , Yuan X , Xiuchun Y , et al. The quality of life of post‐discharge patients carrying drainage tube after PTBD: a qualitative study. J Interv Radiol. 2018;27(02):178‐180.

[21] Yang X , Qin Y , Mo W , et al. Analysis of targeted post‐operative nursing outcome in 1246 patients with percutaneous transhepatic biliary drainage. Front Surg. 2022;9:908909. 10.3389/fsurg.2022.908909 35574558 PMC9094711

[22] Li S , He X , Dang L , et al. Efficacy of ^125^I versus non‐^125^I combined with transcatheter arterial chemoembolization for the treatment of unresectable hepatocellular carcinoma with obstructive jaundice. Dig Dis Sci. 2018;63(2):321‐328. 10.1007/s10620-017-4899-x 29305738

[23] Xia LL , Su T , Li Y , Mao JF , Zhang QH , Liu YY . Improving rehabilitation and quality of life after percutaneous transhepatic cholangiography drainage with a rapid rehabilitation model. World J Clin Cases. 2021;9(34):10530‐10539. 10.12998/wjcc.v9.i34.10530 35004984 PMC8686152

[24] Chayadi E , Baes N , Kiropoulos L . The effects of mindfulness‐based interventions on symptoms of depression, anxiety, and cancer‐related fatigue in oncology patients: a systematic review and meta‐analysis. PLoS One. 2022;17(7):e0269519. 10.1371/journal.pone.0269519 35834503 PMC9282451

[25] Nennstiel S , Weber A , Frick G , et al. Drainage‐related complications in percutaneous transhepatic biliary drainage: an analysis over 10 years. J Clin Gastroenterol. 2015;49(9):764‐770. 10.1097/MCG.0000000000000275 25518004

[26] Ortiz MR . Transitional care: nursing knowledge and policy implications. Nurs Sci Q. 2019;32(1):73‐77. 10.1177/0894318418807938 30798750

[27] Muijsenberg AJL , Houben‐Wilke S , Zeng Y , Spruit MA , Janssen DJA . Methods to assess adults' learning styles and factors affecting learning in health education: a scoping review. Patient Educ Couns. 2023;107:107588. 10.1016/j.pec.2022.107588 36502561

[28] Murugesu L , Heijmans M , Rademakers J , Fransen MP . Challenges and solutions in communication with patients with low health literacy: perspectives of healthcare providers. PLoS One. 2022;17(5):e0267782. 10.1371/journal.pone.0267782 35507632 PMC9067671

[29] Daoqiong H , Xiaoye S , Jun L , et al. The application of feedback health education mode in patients after receiving percutaneous liver puncture and biliary drainage. J Interv Radiol. 2022;31(03):294‐297. 10.3969/j.issn.1008-794X.2022.03.018

[30] Jones SC , Waynick‐Rogers P . An educational tool for promoting transitional health care planning by nurses from the hospital to the community. Nurs Educ Perspect. 2020;41(3):190‐192. 10.1097/01.NEP.0000000000000463 31107810

[31] National Health Commission . National Nursing Development Plan (2021‐2025). Chin Nurs Manag. 2022;22(06):801‐804. 10.3969/j.issn.1672-1756.2022.06.001

[32] Drennan VM , Ross F . Global nurse shortages—the facts, the impact and action for change. Br Med Bull. 2019;130(1):25‐37. 10.1093/bmb/ldz014 31086957

[33] Pek CJ , Van Dijk M , Groot Koerkamp B , Moelker A , van Eijck CHJ . A national survey on peri‐interventional management of percutaneous transhepatic biliary drainage. Surg Laparosc Endosc Percutan Tech. 2017;27(4):253‐256. 10.1097/SLE.0000000000000445 28708769

[34] Miao X , Lihong W , Baojun Y , et al. A survey of PTCD drainage tube nursing in 130 hospitals for malignant biliary obstruction. Chin Gen Pract Nurs. 2020;18(04):489‐491.

[35] Nursing Branch of Cancer Minimally Invasive Treatment Professional Committee of China Anti‐cancer Association, Interventional Perioperative Professional Committee of Interventional Physician Branch of Chinese Medical Association, 15th Radiological Nursing Working Group of Radiology Branch of Chinese Medical Association . Expert consensus on drainage tube nursing of percutaneous transhepatic biliary drainage. Chin J Mod Nurs. 2020;36:4997‐5003. 10.3760/cma.j.cn115682-20200902-05180

[36] Pang Z , Wang X , Wu Y , et al. Construction and empirical study of nursing quality evaluation index system of hepatobiliary surgery based on ERAS concept. Comput Math Methods Med. 2022;2022:1117880. 10.1155/2022/1117880 35465012 PMC9023162

[37] Fang JT , Chen SY , Yang LY , et al. Improving transitional care through online communication skills training. Aging Clin Exp Res. 2022;34(12):3063‐3071. 10.1007/s40520-022-02251-4 36129617 PMC9489478

