# Supplementary material for: Perceptions of transitional care services among patients with percutaneous transhepatic biliary drainage and multicentre health professionals: A qualitative study
Source: Health Expect. 2023 Nov 20;27(1):e13913. doi: 10.1111/hex.13913 (PMC10726261; doi:10.1111/hex.13913)
Supplement: Supplementary file 2 — Supporting information. [file HEX-27-e13913-s006.docx]

# Interview outlines

## The interview outline of PTBD patients (English version)

**Part I: Basic information**

1. **Age: __________** years old
2. **Duration with PTBD drainage tube:__________** month
3. **Educational level:__________**
4. **care mode of PTBD drainage tube:**
5. Home care ;
6. Hospital care ;
7. Community care ;
8. Mix of two modes:**__________**

**Part II: Feelings and perceptions of transitional care service**

1. **How did you feel during the time you wear the drainage tube ?**

(Prompt: Can you talk about any physical condition and psychological feelings during this period?)

1. **What problems have occurred with your drain care after discharge from the hospital? If there was a problem, how did you solve it?**
2. **What transitional care services did you receive from healthcare professionals at the time of discharge and after discharge from the hospital? Are you satisfied?**

(Prompt: Can you talk about how they provided you with discharge instructions? What are the specific aspects that satisfy you?)

1. **What care needs are not being met? How do you want to provide it for you?**

(Prompt: Why do you think so?)

1. **What other feelings and thoughts do you have about transitional care?**

## Interview guide for professional healthcare professionals (English version)

**Part I: Basic information**

1. **Age: __________** years old
2. **Educational level:__________**
3. **Professional title:__________**
4. **Position in the ward:__________**
5. **Years of specialist work:__________**

**Part II: Feelings and perceptions of transitional care service**

1. **Can you describe your role in the transitional care of PTBD patients with tubes?**

(Prompt: What is your job content? Why did you take on this role?)

1. **How do you feel about the quality of transitional care services for PTBD patients with tubes?**

(Prompt: How about the effects of transitional care services of PTBD patients?)

1. **What effect do you think should be achieved in the quality of transitional care for PTBD patients with tubes?**

(Prompt: Which aspect is the most important concern?)

1. **In your opinion, what aspects should be done to further development transitional care of PTBD patients?**

(Prompt: Why do you think so? What do we need to pay attention to？)

1. **What other ideas do you have about transitional care ?**
