# Supplementary material for: Perceptions of transitional care services among patients with percutaneous transhepatic biliary drainage and multicentre health professionals: A qualitative study
Source: Health Expect. 2023 Nov 20;27(1):e13913. doi: 10.1111/hex.13913 (PMC10726261; doi:10.1111/hex.13913)
Supplement: Supplementary file 3 — Supporting information. [file HEX-27-e13913-s003.docx]

Appendix 3

Supplementary table with all quotes of patients’ interview themes

| Themes | Subthemes | Codes | Quotes |
| --- | --- | --- | --- |
| Recognition of transitional care services | Positive attitude of healthcare professionals | Patience and carefulness | *“I am not well educated and I don't understand a lot of things, so when I have problems I call the nurses for help, and after a while I learnt a lot and was able to care for myself proficiently, and I am really thankful to them for being so patient all the time.”* (P2)  *“You always have a smile, and you are very patient in helping me to change the medicine and care, I am very satisfied with your work.”* (P5)  *“They (doctors and nurses) were very professional and careful,* *I hope that under their guidance, I can live better with drainage tubes.”* (P8)  *“I'm fine, I'm recovering quite well now because your doctors and nurses are very responsible.”* (P9)  *“The previous time we phoned to ask about issues with clogged drains, bile leaking, and other things, you guys were incredibly responsible and gave us careful instructions on what to do.”* (P11)  *“You guys did a good job. Every time we've come here, you've always paid close attention.”* (P12) |
|  |  | Timely responses | *“I contacted the medical staff if I had any questions, the replies were quicker and all were answered.”* (P2)  *“I would call Dr Cheng if I had any problems, and he was very happy to help me, because I had an operation at West China Hospital, and I guess I had borrowed hundreds of thousands of dollars up to now, so it was very annoying, and Mr Cheng came to help us when he learnt about our situation.”* (P4)  *“Yes, we could ask questions in the WeChat group and the medical and nursing staff answered us quickly.”* (P5)  *“Usually the doctors and nurses answer when we call, and we know not to bother the doctors when they are busy and can't call.”* (P6)  *“When I was previously hospitalized in another unit, I felt that the doctors and nurses there weren't very motivated; once I messaged them in the morning to inquire about the dosage of my medication, and they didn't get back to me until two or three days later. But you guys just reply very quickly, showing that there is concern about our situation.”* (P10) |
|  | Benefits of transitional care services | Feeling positive about regular follow-ups | *“I know you are also very busy, there are many patients need you to take care of, so I am very touched and satisfied that you still come to care and help us after being discharged from the hospital”* (P3)  *“When the nurses called us after we have discharged from the hospital to show her concern for us and express her wish that we would recover our health soon, and we felt very warm in our hearts.”* (P7)  *“Every time I was discharged from the hospital, you would call to determine how I was doing. With your care and help, I can take better care of myself now!”* (P8)  *“I was sick right and my kids came and took care of me, but I'm still living on my own when I get out of the hospital later on, and my kids are busy with work and their own families, so it is nice that you guys call in to talk with me.”* (P9)  *“The return visit was quite satisfactory, (the nurse) would ask about my condition, see if I needed help, and said to contact them if it got serious.”* (P11)  *“I feel that (continuity of care) is pretty good, I can feel your thoughts and it does help us.”* (P13) |
|  |  | Solving the problems related to self-care | *“I will send a message to the group of drainage tubes if I have a problem, and when the doctor or nurse in charge is available, they will respond. When I run into the same issue again in the future, I'll be able to figure it out on my own.”* (P1)  *“I am not well educated and I don't understand a lot of things, so when I have problems I call the nurses for help, and after a while I learnt a lot and was able to care for myself proficiently, and I am really thankful to them for being so patient all the time.”* (P2)  *“He was very carefully to explain to me where I could go to change the medication and that any of the neighboring clinics would be able to do it. However, when I returned later (because of the epidemic), I was unable to leave the house and had to purchase drains, gauze, adhesive tape, and iodophor online. Then I sterilise myself and change the medicine at home.”* (P3)  *“This service is good because before a lot of things we do not understand, and now with your help, when I encountered some simple care problems I can solve themselves, such as the last time I encountered blocked tube I have successfully resolved it.*” (P4)  “*Unlike in the past when we did not even know where to contact the doctors after discharged, I actually think that this transitional care service is quite good for us patients, giving us a pathway to speak with the doctors and nurses.*” (P6)  *“The previous time we phoned to ask about issues with clogged drains, bile leaking, and other things, you guys were incredibly responsible and gave us careful instructions on what to do.”* (P11) |
| Patients have some unmet needs | Potential psychological needs | Excessive worry about drain prolapse and infection | *“Because I had an open wound on my stomach, I was worried that it would get infected and that the tube would fall out because of my activity.”* (P1)*“I'm worried that by lifting the drainage bag like this, the drainage fluid will back up and cause an infection.”* (P2)  *“The doctor says you can sleep on your side, but I'm still worried that the tubes will come out when I sleep, so I sleep on my back.”* (P3)  *“The doctor said I need a drain to get the bile out and relieve my symptoms, so I'm trying to protect the drain... if it falls out it's going to be a big problem...”* (P6)  *“I was afraid to exercise, and once the drain fell out a few centimeters, I was worried if I had moved and caused the drain to fall out.”* (P8)  *“I haven't even bathed comfortably since I had the tube, and I can only scrub every time for fear of the wound getting infected.”* (P9)  *“I know I have some anxiety and can't help but think about it more (complications from the drain), possibly because of my personality. I tried telling my family about my anxiety, but they advised me not to think about it too much.”* (P12) |
|  |  | Self-image disorders affecting social interaction | *“I wish the doctor would have let me have the surgery sooner because it is inconvenient with the drains you know? There is socialization but it is rare.”* (P2)  *“I rarely left the house and I didn't want people to see my drains and look at me with a measuring or sympathetic look.”* (P5)  *“I rarely go out after the tube is installed, because when people see it, they will say how come you have a drainage bag at the age of 40, are you suffering from some terminal disease?”* (P7)  *“(The drain) affects my socializing, it affects me going to work, but no one has taught me how I should manage this, and more or less the drain affects me a little bit anyway.”* (P9)  *“Many skirts are not convenient to wear after taking the tube, and sometimes I feel embarrassed to go out.”* (P10)  *“I used to frequently go on walks outside, but ever since I had a drain, I have felt embarrassed to do so.”* (P11)  *“At first I felt the impact, but my physical condition is just worse, only resigned to fate, and now I rarely see relatives and friends, it is possible that they know my situation and then greetings.”* (P12) |
|  |  | Irritation with the inconvenience of living with a tube | *“It must have had a big impact on my life in the beginning, and I need to carry the drain for life, so I just have to accept it. But sometimes it's annoying to have to get stitches. I've had hundreds of stitches over the years.”* (P4)  *“Counting, I have had the tube for eight months, I can honestly say that at first it was really unpleasant and uncomfortable. I had to be careful, gradually get used to it, but sometimes I felt upset. I hope that the drainage tube will be removed soon.”* (P6)  *“Every time I put on and take off my clothes, it's very troublesome because I am afraid of tearing off the drain tube, and I usually sleep on my left side only, because I am worry about pressing on it if I sleep on my right side.”* (P7)  *“I can not take a shower, I can only take a towel and dip it in water to scrub my body, it is very hot in the summer and I have to scrub it many times, so how can you say it is not annoying?”* (P8)  *“I haven't even bathed comfortably since I had the tube, and I can only scrub every time for fear of the wound getting infected. I also don't dare to eat spicy or stimulating things, but we Chongqing people just love spicy food. (annoyed)”* (P9)  *“It was painful at first with the tube, but now I'm used to it after 6 months, but I feel quite annoyed with the drain sometimes, so I tell myself to hold on a little longer!”* (P10)  *“Sometimes it's the tape that makes me feel itchy and I want to scratch it, but I'm afraid I'll get the tape off, and that makes me feel a little uncomfortable.”* (P11)  *“Sometimes it is quite annoying to have a drain, I can not shower comfortably, I have to sleep more carefully, and I go out less often.”* (P13) |
|  | Knowledge needs for disease care | Management of drainage tube emergencies | *“One time my drain fell out, but the doctor probably did not think about that and did not tell me, so when the tube fell out, I put it in myself and it hurt my stomach.”* (P1)  “*The doctor only mentioned changing the medication and drainage bag on a regular basis; no particular precautions were mentioned. When I was released from the hospital, it was still hot in August.It seems that you can change the bag a little bit more frequently when the weather is hot; in any case, just change the medicine and the wound.* ” (P3)  *“When blood-colored fluid appeared in the drain, I was worried if I was getting worse, and all I could do was ask the doctors and nurses for help.”* (P4)  *“The doctor said it's normal to go back in a week or two, but I don't know if I need to wear this drain for a long time, I have a brace, but I don't know what the purpose of these things is. Will the brace and drain be affected when I bend over or exercise? Is it made of plastic or what kind of material, will it hurt if I press on it, or will it fall off?”* (P5)  *“My gauze would often get soaked and I wouldn't know what to do with it, then I asked my doctor and was told to change it regularly.”* (P6)  *“You should monitor the drainage fluid, safeguard the drainage tube, return to change the medicine carefully, and change the drainage bag frequently, said the doctors and nurses. But I also want to know what I'm going to do if something happens to the drain. What should I do if I can't reach a doctor or nurse?”* (P7)  *“I noticed that my drains were often clogged, and after consulting with my doctor he taught me that I had to go and squeeze the drains on a regular basis. If he had taught me how to deal with it at the beginning when I was discharged from the hospital, I would not have panicked so much after I was discharged from the hospital when I encountered the problem, and I wouldn't have bothered them (doctors and nurses) so often.”* (P8)  *“They (the nurses) taught me to change the drainage bag in 7-10 days and to protect the drain, but not much else. Usually the doctor talks, then I listen and also want to learn more about the disease, drain care, etc. So that if I encounter any problems, I can solve them myself. I live on my own, I don't live with my children and I have no one to help me if something happens”* (P9)  *“Once I got up in the middle of the night to go to the bathroom, I was not very lucid and I just felt something tugging at me, so I simply took it out. It turned out to be my drain and I hurried it back in. When my kids took me to the hospital the next day to take care of it, the doctor told me to be careful and not to simply shove it back in after it fell out.”* (P13) |
|  |  | Personalized lifestyle guidance (diet, exercise) | *“The diet is definitely light, not spicy, and I have a stone-related disease, so it's recommended to eat less offal and greasy food. The doctor also told me not to exercise strenuously, but I'm not really sure what kind of exercise is appropriate, and I'm worried that the drain will fall out because of the exercise.”* (P1)  *“I heard that Gallstones are prone to relapse, and I hope to learn more knowledge about diet to prevent diseases. It's often said about these types of diseases that it's important to eat the right foods”* (P2)  *“The nurse did say to eat a light diet normally and did not seem to say much else. But I have diabetic, and I am wondering if there are any changes in my diet now that I am wearing the drains.”* (P3)  *“I know how to eat a light diet, not very greasy. I also need to change the gauze, and for exercise, like me, I can only walk slowly, not run. I would like to know if there is anything else I should be aware of in my diet regarding my condition”* (P4)  *“When we were discharged from the hospital usually the nurses talked to us about the precautions to be taken, vaguely said that the diet should be light and regular, eat fewer meals and more frequent meals.”* (P5)  *“Exercise is not particularly stressed, anyway, just pay attention to yourself, so I am looking for more professional advice in this area.”* (P8)  *“Because now my liver function is compromised, but I am unsure what foods I can and can not eat in my diet, and the healthcare professionals also do not seem to have been very specific talked about this.”* (P11)  *“I do not eat well, I can feel I am mental poor, and I find it is difficult for me to eat a lot. I think I need some dietary guidance from the doctors.”* (P12) |
| Choice of transitional care service pathways | Preferred transitional care model for the hospital-to-home transition | Savings in time and money | *“We can change the medicine in our community and also in the neighbouring clinics, mainly to sterilise and dress the wounds, which I can actually do myself at home and save more money.”* (P1)  *“A few years ago when I had a drain, I would go to the community to change my medication and it was only 6 RMB per change, but now it's too expensive! A change of medication now costs a few tens of yuan, so now I usually change my own drainage bags and medication at home, and I only go to the community hospital when the drain is a little loose and needs stitches to hold it in place. I have had hundreds of stitches over the years and it is cost me almost 30 yuan every time.”* (P4)  *“I prefer that doctors and nurses teach me how to care for the pipes, because my home is far away from the town, and it costs some money every time.”* (P5)  *“After learning to change the drainage bag, I will change it by myself, and there is no need to run around. I will consult the doctor or nurse if I don't understand.”* (P7)  *“I usually take care of drain tube by myself because it is a hassle to change the medicine. My house is far away from the hospital, my house is in the countryside, if you go to change the medicine once, it will cost you 10 or 20 yuan, plus you will have to spend tens of yuan on the bus fare for the trip, so I change it myself at home.”* (P8)  *“We usually change our own medication at home, for example, if the gauze on my wound gets soaked with bile, we buy gauze online to change it, and nowadays everything is cheap online.”* (P9)  *“It is cheaper and more comfortable to transition to living at home after being discharged from the hospital, after all, you are not confined to your own home.”* (P13) |
|  |  | Availability issues with drainage tube care services | *“(The way in which transitional care is provided) depends mainly on the convenience, if I have to travel a long way, then I would find it a bit of a hassle.”* (P5)  *“We did go to find out if the community hospital near our house could care for the drain, but it turns out they don not have that service at the moment.”* (P9)  *“Sometimes I would go to the community to care for the drains, but when the epidemic was still worse, we changed the medication at home by ourselves and my husband changed it for me, he knew how to do it after seeing the doctor a few times before, so he changed it for me at home.”* (P10)  *“The doctor meant to say to go to a clinic to change the medicine, but when we got to those clinics, the doctors at the clinics said that they did not have that kind of gauze and the clinics would not change it. Later, they went to the Internet to look up the relevant information and bought the gauze themselves to change it for us.”* (P11)  *“I went to the community hospital for cold and flu those, but not for drain care because the community doctor said they did not know my condition and the wound and were afraid to deal with it for me.”* (P12) |
|  | Receiving network information guidance and telephone follow-up | Convenient and swift | *“Sometimes, despite the distribution of brochures, we may lose them, but this is not the case with the web-based approach.”* (P1)  *“I think it is (network information guidance) great that we can learn how to care for drains by watching videos, and even if we forget, we can watch them repeatedly to avoid bothering the physicians and nurses as frequently.”* (P4)  *“I quite support the use of platforms such as WeChat to carry out transitional care services because nowadays everyone cannot live without a mobile phone, and if one does not know how to access the Internet, then one can make a phone call, and it is all very convenient.”* (P5)  *“In fact, a phone call would be fine, a week or half a month after I am discharged from the hospital, you guys call to care about how I am doing, and I don't think that would be too intrusive for my life.”* (P7)  *“We happened to be talking about this today, and I think the online information guide is quite good, we can access services quickly and it is convenient to talk about any questions.”* (P8)  *“You can use the internet, we can learn on it, it is all very convenient now, otherwise we will forget after you have talked about it.”* (P9)  *“I think WeChat official account would be better, we would all follow it, young people basically rarely read paper these days, I mean honestly.”* (P10)  *“It feels like it is a digital age now, and things like registering, doing tests, etc. can be accessed on mobile phones, so I think it's easier to continue care through the internet.”* (P11)  *“After all, we are not professionals, and I just wish we had a convenient way to learn more about disease-related issues.”* (P13) |
|  |  | Network guidance visibility | *“I am an elderly lady now, and I rely largely on my kids about the internet, but I still think this (network information guidance) is a good strategy, because in this way we can learn visually.”* (P3)  *“I think we need to "see" in order to learn, especially for the operation like drain care, and it is difficult to grasp this part of knowledge just by listening to the dictation.”* (P5)  *“In this way, the knowledge is also comprehensive and we are able to understand the content better, and if there is something we don't understand, we can also contact our healthcare provider.”* (P6)  *“This is a convenient way for us to learn and to share our knowledge on disease prevention with our family and friends as we browse through it.”* (P8)  *“I feel like it is more informative by browsing through videos or tweets. It was easier to understand for someone like me who is not well educated.”* (P11)  *“I feel that the user interface of network guidance is relatively clear, and we can quickly find the services we need, which is very helpful.”* (P12) |

Supplementary table with all quotes of healthcare professionals’ interview themes

| Themes | Subthemes | Codes | Quotes |
| --- | --- | --- | --- |
| The harvest and challenges of transitional care services | Creating a positive emotional experience | Appreciation and trust from the patients | *“When I provide health education to patients, I will tell them very graphically that bile is like vegetable oil, it's a clarified, dark green liquid. If you find blood-colored, or purulent conditions in it, and you are accompanied by abdominal pain, then you must come to the hospital. The patients then felt that my explanations were clear and said that I was a good teacher.”* (N2)  *“Because I am mainly engaged in nursing management type of work, I do hear from patients or their family members that we have done a good job in this area and that I should praise our nurses.”* (N3)  *“A patient wrote me a thanks letter before. In fact, these are what I should do, but her sincerity touched me. I will always remember this trust and strength.”* (N4)  *“When we succeed in helping patients deal with the unexpected situations they encounter, the patients are really grateful (to us) because they are actually very helpless when they encounter problems.”* (N5)  *“Not only do we have to instruct our patients in self-care, but we also have to do a good job with their families, and it's important to communicate effectively, so generally speaking, we have a pretty good relationship with our patients.”* (N6)  *“I was touched by the rapport I had previously established with the patient through my care and assistance while the patient was in hospital, and continued follow-up after discharge.”* (N9)  *“A previous patient's family was in a difficult financial situation, and after she was discharged from hospital I tried to help her adapt to home care as much as I could, so she was very grateful to me. I have heard the words of thanks many times, but I am still touched by the patient's sincerity!”* (N10) |
|  |  | Improvements in drain care outcomes | *“I think it makes me happy to be able to help patients and to see that they are able to take good care of themselves even after they are discharged from the hospital, which shows that my work is worthwhile.”* (N1)  *“We also hold a symposium every month, which is equivalent to selecting family members or patients with drainage tubes and having a group education on knowledge, this is also very helpful for drain care for patients”* (N2)  *“I have taken on the role of missionary and mentor in my work, and I feel that I have realized the value of my work through my own efforts.”* (N3)  *“In fact, as doctors, we are problem solvers for patients, and we can also feel that through the transitional care service, our patients are much better when they come to the hospital the next time.”* (N4)  *“Because there are a lot of patients with drains like this in this area, and usually they may be discharged soon after the puncture and placement of the tube, our transitional care can help the patient to better adapt to life with the tube.”* (N5)  *“Our existing transitional care model can help patients make a smooth transition to home, and from patient feedback, I think the results are effective.”* (N7)  *“With the help of transitional care, patients did not have pipeline complications, were satisfied with our medical care services, and were able to achieve early extubation.”* (N8)  *“In fact, the need for continuity of care in this area is very great, because we have a long period of time after surgery for surgical patients, like post-surgical diet, activities, all aspects of the quality of life need to be a supervisory.”* (N11)  *“I mainly take on the role of disease treatment, but in fact the time the patient spends at home recuperating is also crucial for subsequent treatment, and the results show that transitional care fulfils its role.”* (N12) |
|  |  | The improvement of one’s professional ability | *“When instructing patients, we make an effort to use as many simple terms as we can to make sense to them. You must be skilled at telling and know it inside and out at this point.”* (N2)  *“When a patient comes to us for help outside the hospital, we need to make a quick response strategy according to the patient's situation, which is actually a great test of our ability.”* (N5)  *“Through serving patients, I have also learnt how to communicate better with them, which also helps me in interpersonal communication in my life.”* (N6)  *“In the extended care service I need to answer the patient or family's questions, then I should first of all be very aware of the process and knowledge myself, it will promote me to learn, I will also listen to the doctor's instruction to them on the side, and it is also a kind of improvement for myself.”* (N9)  *“There were patients who didn't quite understand drain care and post-discharge medication at first, and through our health education, they learnt more, and I gained professional satisfaction from it.”* (N10) |
|  | Existing difficulties and challenges | Insufficient human resources for healthcare professionals | *“It is true that we are very busy with our work, with limited energy, and sometimes we can not do as much as we would like to do about a lot of things.”* (N1)  *“I think the most important problem is human resources, with more human resources can solve other problems.”* (N2)  *“It's true that doctors and nurses are very busy, and I schedule a lot of surgeries, and I have limited energy right now, and it's usually the patient who initiates contact with me, and it's rare that I'll go in on a regular basis to care about how his drains are doing and all of that, and that's seldom done.”* (N4)  *“Because right now we're all very busy in our section at work, and everyone is very busy at work. After going back from work we all feel that we don't have the energy to deal with this matter.”* (N5)  *“There is also the issue of our human resources, because you want to do this thing, information technology is not enough, you have to have a dedicated person to manage, and the requirements for the management of the person he is a little bit high, I think he has to be more specialised, some of the diseases of the undergraduate department, all aspects of it should be very clear, the patient asked what he can be able to respond quickly, so that kind of his ability to require is very high. I think it's really a specialised person who can do this in order to do it well and extend it.”* (N7)  *“We are now very busy at work, and now we are mainly arranging for a dedicated person to follow up, so we have not fully implemented the idea of letting our own charge nurses to implement the follow-up visits, and the charge nurses are mainly responsible for the work of discharge instructions in transitional care.”* (N8)  *“Human resources are a big problem, there is a lot of work every day, and sometimes I feel as if it is almost enough to do a good job in the basic operations for patients. Transitional care needs to spend more time and energy to educate and contact patients.”* (N9)  *“I think the main difficulty is human resources, right? I don't think those are problems, as long as there are people, those problems can be solved.”* (N10) |
|  |  | Process and education guidance need to be optimized | *“Transitional care services can be problematic if there is not a very standardised service process in place, and with the appropriate service process in place, it's easier for us to follow it."* (N1)  *“Sometimes the patient will ask us what they should do to change the drainage tube themselves, and we will guide them. However, we don not have uniform guidelines, and if you do not do a good job, you might get in trouble.”* (N2)  *“There is not yet a clear evaluation system, we will judge by patient satisfaction and complication rate, unplanned admission rate, etc., but in fact it is not very clear how well the measures of transitional care for nurses have been implemented.”* (N3)  *“Usually patients are discharged from hospital for a period of time and need to return to hospital for the next step in their treatment, and if we can deepen the content of our transitional care, I think it would be very helpful for the patient's next step in their treatment.”* (N4)  *“We found that the current transitional care in the department is done in a very general way and none of it is very detailed. For example, this patient's dietary mission, the specific time of the follow-up visit, and the time of the next drainage bag change and the specific time of the patient's return to the hospital were not reflected.”* (N5)  *“Transitional care is carried out relatively late, health care workers are unfamiliar with its service process, there is a need to have a set of standardised and complete process before health care workers can go to provide services to patients.”* (N7)  *“We must teach our patients and families what is right and not what is wrong or it will affect the trust of the nurses.”* (N8)  *“We are currently doing both oral instruction and WeChat official account missionary work, but it doesn't feel like it's a very complete thing yet.”* (N10) |
|  |  | Lack of support from a well-developed information system | *“Some PTBD patients carry drains for long periods of time, which also puts a higher demand on the data storage capacity of the transitional care platform.”* (N2)  *“The current situation is that there is also a disconnect between doctors and nurses with respect to some of the patient's information, because doctors and nurses use different management systems.”* (N3)  *“As far as I know, there is no information system to interface between hospitals and the community, and community hospitals are unable to know the conditions of their patients, so it is felt that more patients are willing to opt for home care.”* (N4)  *“If we want to manage patients more systematically, the current transitional care platform really feels like it needs to be further improved and the functions and services of the platform need to be refined.”* (N5)  *“In fact, I was originally thinking of a problem, that is, the extent of our informatisation, which feels that it is still at a relatively basic stage, without highlighting the needs of patients.”* (N7)  *“In fact, I think the information system should be opened up. From the perspective of a doctor, it is important to achieve the flow of information, so as to understand the situation of the patient and facilitate a better response when the patient seeks help.”* (N11)  *“It is good to have a WeChat platform, so that patients can ask questions directly, and we can push nursing knowledge, but if I want to do more, such as monitoring the patient's postdischarge diversion, the WeChat group chat approach dose not work.”* (N12) |
| Expectations for future development | Hospital and departmental level | Human resource allocation and training | *“There is a need for more training for charge nurses in the future as they work directly with patients and have many opportunities to educate patients and families.”* (N2)  *“I feel that at least in each of our wards, there are separate people who co-ordinate the management of patients' transitional care and invest their time and energy.”* (N4)  *“I feel that there is a need for members with stronger communication and management skills to guide the development and improvement of transitional care, as well as to enhance communication between doctors and nurses.”* (N5)  *“Human resource allocation and staff training need to be strengthened, and perhaps we need to develop case managers as well.”* (N7)  *“It is important to build multidisciplinary teams, where in addition to members with medical backgrounds, information professionals we may also include, and where members need to actually fulfil their roles, not just a formality.”* (N8)  *“The human resources aspect needs to be considered, and training is also an important aspect. More people need to be involved in the future, it is not something that can be done by one person”* (N9)  “*It is best to have someone responsible for coordination, which can take the form of a team, and doctors and nurses should also strengthen communication and training.”* (N11) |
|  |  | Support from facilities and related norms | *“Now when it comes to patient safety, you have to be rigorous. That's why our norms need to be rigorously developed and enforced.”* (N1)  *“My idea was to refine each aspect of PTBD patients' transitional care instructions, such as drain care and instructions on diet and exercise, to be more informative and specific, with pictures or in a more creative video format, as many patients are still unclear about these aspects.”* (N2)  *“The transitional care norms you develop should be scientifically based, or patients may question you. Sometimes we really meet a very critical patient who again does not understand something will go and find a lot of information and then come and discuss it with you.”* (N3)  *“Or we should start transitional of care as early as possible, then we should first define a reasonable transitional of care process and norms. For example, we can increase the number of patient follow-up visits.”* (N4)  *“We must review the literature, develop scientific guidelines, improve follow-up systems and processes, and pass on the right knowledge to patients and families.”* (N5)  *“In the future, we should optimise the system and norms of transitional care services, and nurses should pay attention to assessing whether the patients have accepted it and to what extent they have accepted it when they carry out the education. Because we are facing patients with different levels of education, age and disease conditions.”* (N7)  “There are new models of integrating case management into continuity of care, and the work is being done with the support of information platforms.” (N8)  *“Wechat public accounts and small programs can be used to release some education videos for patients to learn and watch.”* (N9)  *“I hope that we can introduce a smarter platform to help us build personal health records for discharged patients and implement personalized management.”* (N10)  *“We can seek help from unit directors and nurse managers to improve our existing transitional care platforms, and although more manpower and resources are invested, this will actually yield a lot of invisible rewards.”* (N11)  *“If the platform can realize the regular push and monitoring function, but also does not involve the privacy of the personal account of the health care that would be better, some health care or more mind, like they will not leave their own private phone.”* (N12) |
|  | Social level | Anticipation of community participation | *“I think in the future there is still a need for more cooperation and exchanges between tertiary hospitals and community hospitals or lower level hospitals.”* (N1)  *“I think it is important to work with the community because the general trend is that more and more patients are being discharged with tubes. If the community develops, then the resources of the large hospital can be properly allocated, and patients can save time and energy.”* (N2)  *“The knowledge level of community workers is relatively weaker, and we can provide them with regular training or go to guide their work. Because they manage a limited number of PTBD patients, we can also provide telephone or video guidance when the community needs assistance.”* (N3)  *“I think it is very meaningful. Some large hospitals, especially those with a particularly large number of patients with drains and a rapid turnover, especially need to cooperate with lower-level hospitals or the community to carry out transitional care, which can save the patient's length of stay and costs, and increase the convenience of his access to health care, which can enhance his satisfaction.”* (N4)  *“Because nowadays the country's healthcare cannot say that all patients go to tertiary and secondary hospitals. Because this leads to a lot of pressure on medical care.”* (N5)  *“Community workers have previously mentioned that their limited experience and selection of dressings make it challenging for them to manage patients who have infections after carrying drains. They can then transferred patients to us at this time.”* (N6)  *“It is definitely better if we can work with the community, for example, if we encounter a minor problem, such as a patient changing a dressing, fixing a dressing or drainage bag, then it would be more convenient to change it (change medication, etc.) at a nearby community hospital. When there is a emergency sometimes need to be dealt with, for example, if a drain has come off accidentally, the community can deal with the patient first and stabilise the patient's condition.”* (N7)  *“First of all, you need to pay attention to the difference between large communities and small communities, perhaps the level of personnel in the health service stations in large communities is slightly higher, and then he will be willing to take on these things, willing to help and willing to do it, and they feel that there is a sense of significance as well, and this work may be able to be carried on.”* (N8)  *“Ideally it would be the local community or hospital that also manages this, they can do some of the work and patients with problems are dealt with directly close by.”* (N11)  *“Our experience is different from that of community workers. Sometimes it is very tired to communicate with them. Therefore, limited by knowledge, we need to strengthen training.”* (N2)  *“The problem we are facing is now that we have some medical joint units, the patients are relatively scattered, and many units have not established contact with them. If we can centralize in certain community hospitals now, for community workers, and then we can communicate among doctors to create a group and communicate in it, it will be very good to protect the privacy of patients.”* (N4)  *“If we are going to work together, we definitely need to communicate with each other to understand and sign the appropriate paperwork, or do the appropriate training and so on, which I think may be necessary.”* (N7)  *“In fact, we can go to the community in Chongqing to link up because it does help with transitional care, but it needs support from the top to the bottom, and it's not something that can be accomplished by a particular person; it needs more people to consider the issue before the community will pay attention to it.”* (N9)  *“We hope to work with the community to reduce the pressure, but we also need to find out whether community hospital workers are willing to participate in the transitional care services for patients with PTBD, and the whole work will require a great deal of effort if it is to be completed and carried out.”* (N12) |
