# Supplementary material for: Perceptions of transitional care services among patients with percutaneous transhepatic biliary drainage and multicentre health professionals: A qualitative study
Source: Health Expect. 2023 Nov 20;27(1):e13913. doi: 10.1111/hex.13913 (PMC10726261; doi:10.1111/hex.13913)
Supplement: Supplementary file 4 — Supporting information. [file HEX-27-e13913-s004.docx]

Example of the coding tree.

| **Participants’ description** | **Original Code** | **Collated extracts** | **Theme** |
| --- | --- | --- | --- |
| *‘I rarely go out after the tube is installed, because when people see it, they will say how come you have a drainage bag at the age of 40,are you suffering from some terminal disease?’* | Drainage tube affects going out | Self-image disorders affecting social interaction | **Patients have some unmet needs** |
| *You always have a smile, and you are very patient in helping me to change the medicine and care, I am very satisfied with your work.* | Attitude affects satisfaction | Appreciate the positive attitudes of healthcare professionals | **Recognition of the value of transitional care services** |
| *I think it's important to work with the community because the general trend is that more and more patients are being discharged with tubes. If the community develops, then the resources of the big hospital can be properly allocated and patients can save time and energy.* | Communities play an important role | It is meaningful to cooperate with the community | **Expectations for future development of transitional care services** |
| *With the help of transitional care, the patient did not have pipeline complications, was satisfied with our medical care services, and was able to achieve early extubation.* | The effect of transitional care | Improvement of patient satisfaction and nursing outcome of drainage tube | **Harvest and challenges in transitional care services work** |
