# Supplementary material for: Perceptions of transitional care services among patients with percutaneous transhepatic biliary drainage and multicentre health professionals: A qualitative study
Source: Health Expect. 2023 Nov 20;27(1):e13913. doi: 10.1111/hex.13913 (PMC10726261; doi:10.1111/hex.13913)
Supplement: Supplementary file 6 — Supporting information. [file HEX-27-e13913-s005.docx]

Supplementary file 2: Example of the coding tree.

| **Participants’ description** | **Code** | **Collated extracts** | **Theme** |
| --- | --- | --- | --- |
| *‘I rarely go out after the tube is installed,because when people see it,they will say how come you have a drainage bag at the age of 40,are you suffering from some terminal disease?’* | Drainage tube affects going out | Fear of social interaction | **Patients with heavy psychological burden** |
| *‘One time my drain fell out,but the doctor probably didn't think about that and didn't tell me,so when the tube fell out I put it in myself and it hurt my stomach.* | Drainage tube emergency treatment | Inadequate and specific guidance at discharge | **Poor management of transitional care at this stage** |
| *I think it's important to work with the community because the general trend is that more and more patients are being discharged with tubes.If the community develops,then the resources of the big hospital can be properly allocated and patients can save time and energy.* | Communities play an important role | It is meaningful to cooperate with the community | **The way of transitional care** |
| *With the help of transitional care,the patient did not have pipeline complications,was satisfied with our medical care services,and was able to achieve early extubation.* | The effect of transitional care | Focus on patient satisfaction and nursing outcome of drainage tube | **Improvement and evaluation of transitional care services** |
